# Biological Aggressiveness of Subclinical No-Mass Ductal Carcinoma In Situ (DCIS) Can Be Reflected by the Expression Profiles of Epithelial-Mesenchymal Transition Triggers

**DOI:** 10.3390/ijms19123941

**Published:** 2018-12-07

**Authors:** Bartlomiej Szynglarewicz, Piotr Kasprzak, Piotr Donizy, Przemyslaw Biecek, Agnieszka Halon, Rafal Matkowski

**Affiliations:** 1 Breast Unit, Department of Surgical Oncology, Lower Silesia Oncology Center, 53-413 Wroclaw, Poland; rafal.matkowski@umed.wroc.pl; 2 Department of Oncology, Faculty of Postgraduate Medical Training, Wroclaw Medical University, 53-413 Wroclaw, Poland; 3 Department of Breast Imaging, Lower Silesia Oncology Center, 53-413 Wroclaw, Poland; kasp@poczta.onet.pl; 4 Department of Pathomorphology and Oncological Cytology, Wroclaw Medical University, 53-413 Wroclaw, Poland; chir.onk.2@dco.com.pl (P.D.); ahalon2@gmail.com (A.H.); 5 Faculty of Mathematics and Information Science, Warsaw University of Technology, 00-662 Warsaw, Poland; przemyslaw.biecek@gmail.com

**Keywords:** ductal carcinoma in situ, epithelial-mesenchymal transition, N-cadherin, SPARC, Snail1

## Abstract

Epithelial-mesenchymal transitions (EMTs) have been recently implicated in the process of cancer progression. The aim of this study was to assess how the preoperative expression patterns of EMT biomarkers correlate with the risk of postoperative invasion in ductal carcinoma in situ (DCIS) found on stereotactic breast biopsies. N-cadherin, Snail1, and secreted protein acidic and rich in cysteine (SPARC) immunoreactivity was observed in 8%, 62%, and 38% of tumors, respectively. Snail1 and SPARC expressions were significantly related to N-cadherin expression and to each other. The postoperative upgrading rate was associated with a positive preoperative expression of all biomarkers. Significance of Snail1 and SPARC persisted in multivariate analysis, but the impact of SPARC on invasion was more significant. When these two EMT triggers were considered together, the risk of invasion did not significantly differ between the subtypes of DCIS with single positive expression (SPARC−/Snail1+ vs. SPARC+/Snail1−). However, it was significantly lower in single-positive DCIS when compared to lesions of a double-positive profile (SPARC+/Snail1+). Moreover, there were no cases in the double-negative DCIS (SPARC−/Snail1−), with foci of infiltrating cancer found postoperatively in residual postbiopsy lesions. In contrast, DCIS with a combined high SPARC and Snail1 expression (intermediate or strong) had an invasive component in 66–100% of tumors.

## 1. Introduction

Since the implementation of mammographic screening programs, the incidence of early breast cancer, in situ lesions, and borderline proliferations has increased dramatically [1]. Among them, ductal carcinoma in situ (DCIS), which is usually diagnosed during a stereotactic biopsy of suspicious microcalcifications detected on a mammography, accounts for approximately 20% of all breast cancers [2,3]. Moreover, studies of the natural course of DCIS managed with biopsies only (no curative therapy) suggest that up to 75% of patients do not develop an invasive disease during follow-up after 15–25 years [4,5,6]. Thus, optimal management and clinical decision-making in newly diagnosed DCIS cases is nowadays a considerable challenge, and the need for a biology-based treatment strategy is postulated [1]. Identification of molecular and biological markers can help to tailor a more individual approach, avoid overtreatment in indolent DCIS, and improve outcomes in aggressive subtypes.

Epithelial-mesenchymal transition (EMT) is a dynamic process whereby an immotile epithelial cell temporarily changes its morphology to a spindle-like shape and acquires the migratory and invasive properties of a mesenchymal cell. This allows for invasion through the basal membrane as well as the extracellular matrix and, ultimately, migration to distant sites, where it reverts back into the epithelial phenotype [7]. EMT is implicated in a number of different biological processes, including embryonic development, tissues repair, and wound healing. EMT has also been shown to play a crucial role in cancer progression, invasion, and metastasis [8]. Molecular changes during EMT are regulated by several transcription factors called EMT-inducers (Snail1, Snail2, ZEB1, ZEB2, TWIST, FOXC2, E47, TCF4), regulators of the extracellular matrix that facilitate the degradation of the basal membrane and surrounding tissues (SPARC, TGF-β, EGF, FGF), as well as molecules from related pathways (e.g., MAPK, P13K, Wnt, NF-κB, Notch, Hedgehog) [9]. Epithelial cells are immobile due to the well-constructed cytoskeleton and the strong cell-cell and cell-extracellular matrix adhesions, which consist of adherens junctions, tight junctions, and desmosomes [9]. To initiate a cell phenotype interconversion, key components of intercellular junctions such as E-cadherin, claudins, occludins, and desmosomes are downregulated by EMT inducers. Loss of E-cadherin expression and the concomitant upregulation or de novo expression of N-cadherin, referred to as a cadherin switch, is considered a hallmark of EMT [10].

In our previous papers, we reported a significant association between the presence of final invasion following surgery in DCIS, diagnosed with a stereotactic vacuum-assisted biopsy as a pure in situ lesion, and a preoperative expression of molecules that trigger the early steps of EMT: Transcription factor Snail1, a direct E-cadherin repressor, and the multifunctional de-adhesive protein SPARC (secreted protein acidic and rich in cysteine), a key modulator of the tumor microenvironment.

The aim of the present study was to investigate whether the predictive value of individual SPARC and Snail1 expression could be improved by the additional assessment of N-cadherin as well as by the complex analysis of the combined expression profiles of the aforementioned EMT biomarkers.

## 2. Results

### 2.1. Clinical Features

Baseline characteristics are presented in Table 1. Among the clinical and pathological variables, there was a significant correlation between the type and distribution of microcalcifications as well as between the nuclear grade and comedonecrosis, as we reported previously [11,12]. Casting-type microcalcifications most commonly presented a regional distribution pattern. Conversely, powdery and crushed stone-like microcalcifications exhibited a clustered and grouped distribution pattern, respectively (*p* < 0.0001). Traditional histological analysis revealed a direct relationship between the presence of comedonecrosis and the severity of the lesion’s grade. A stepwise decrease in the incidence of comedonecrosis could be observed from high (91%) through intermediate (56%) to low (45%) grade lesions (*p* < 0.0001).

### 2.2. EMT Biomarkers in Preoperative Pathology (Biopsy Specimens)

In this study, 8% (17) of DCIS was positive for N-cadherin. Snail1 expression was found in 62% of lesions (129). Positive immunoreactivity was weak, intermediate, and strong in 27% (57), 21% (43), and 14% (29) of cases, respectively. SPARC upregulation was observed in 38% of tumors (79). Positive staining was weak, intermediate, and strong in 16% (33), 10% (20), and 12% (26) of patients, respectively. N-cadherin expression was significantly related to the distribution of microcalcifications (positivity most common in clustered <1 cm, least in regional >2 cm; *p* = 0.033). As we presented before, the correlation between SPARC and the type of microcalcifications was very close to the significance limit (positivity most common in casting type, least common in powdery; *p* = 0.061) [11]. Expression of Snail1 was also significantly associated with the morphology of microcalcifications (similarly to SPARC, most common in casting type, least common in powdery; *p* = 0.041). However, it was also significantly related to the presence of comedonecrosis (72% of Snail1-positive tumors in comedo-DCIS, 48% in noncomedo; *p* = 0.001) [12]. No other significant correlation between the investigated biomarkers and the clinical and pathological variables was observed. Among EMT factors, upregulation of N-cadherin was significantly associated with Snail1 and SPARC expression (*p* = 0.019 and *p* = 0.017, respectively). Positive Snail1 and positive SPARC were related to each other with a very high significance (*p* < 0.0001). The correlation is presented in Table 2.

### 2.3. Final Invasion in Postoperative Pathology (Surgical Specimens)

Invasion was present on final pathology in 34 patients, which gave a postoperative upgrading rate of 16%. Patient age did not significantly influence that risk. Neither mammographic appearance of microcalcifications (distribution, morphology) nor traditional pathologic features (nuclear grade, comedonecrosis) had significant predictive value. Upgrading to invasive cancer was more common in N-cadherin-positive DCIS (35.3% vs. 14.6%). The difference was very close to the significance limit (*p* = 0.0608). The risk of final invasion was significantly higher in Snail1-positive DCIS compared to Snail1-negative DCIS: 25% versus 3%, respectively (*p* < 0.0001). Among Snail1-positive tumors, there was a stepwise increase in the upgrading rate from poor immunoreactivity (9%) through intermediate (26%) to strong staining (55%). The correlation was of very high significance (*p* < 0.0001) [12]. The risk of final invasion was also significantly enhanced in SPARC-positive DCIS when compared to SPARC-negative DCIS: 38% versus 3%, respectively (*p* < 0.0001). Likewise, for Snail1, a stepwise risk increase from poor immunoreactivity (3%) through intermediate (50%) to strong staining (73%) was observed. That association was also very significant (*p* < 0.0001) [11].

Multivariate analysis demonstrated that both Snail1 and SPARC were significant and independent predictive factors for postoperative invasion (*p* < 0.001 and *p* < 0.0001, respectively). However, the impact of SPARC upregulation on invasion was more significant than Snail1 expression: Odds Ratio 3.940, 95% Confidence Interval 2.502–6.637, versus 2.143, and 1.248–3.822, respectively. The association between preoperative features of DCIS and the presence of invasive disease on final pathology is presented in Table 3.

When these two EMT triggers were considered together, the risk of invasion did not significantly differ between the subtypes of DCIS with single positive expression of either SPARC or Snail1 (SPARC−/Snail1+ vs. SPARC+/Snail1−: 6.25% vs. 14.29%, *p* = 0.306). However, the risk of invasion was significantly lower in single-positive DCIS when compared to lesions of a double-positive profile (SPARC+/Snail1+: 7.7% vs. 43.1%, *p* < 0.0001). This significant difference persisted when the subtypes of single-positive DCIS were compared to double-positive DCIS separately: SPARC−/Snail1+ versus SPARC+/Snail1+ (6.25% vs. 43.08%, *p* < 0.0001) and SPARC+/Snail1− versus SPARC+/Snail1+ (14.3% vs. 43.08%, *p* = 0.044), respectively. Moreover, DCIS with a combined high SPARC and Snail1 expression (intermediate/strong) had an invasive component in 66–100% of tumors. In contrast, there was no case (0/66) among the double-negative DCIS (SPARC−/Snail1−), with foci of infiltrating cancer found postoperatively in the residual postbiopsy lesion. Invasion rates are presented in Table 4.

## 3. Discussion

A cadherin switch facilitates the release of cancer cells from the primary tumor, resulting in increased migratory and invasive behavior [13]. We observed that the expression of N-cadherin was more common in DCIS postoperatively upgraded to invasive cancer compared to lesions without final invasion. ElMoneim and Zaghloul found upregulation of N-cadherin in 52% of invasive ductal cancers. It was significantly related to larger tumor size, positive nodal status, higher clinical disease stage, a poor Nottingham prognostic index, and negative estrogen receptor (ER) and progesterone receptor (PR) status. Pure invasive carcinomas were slightly more commonly, but without significance, N-cadherin-positive than those with an intraductal component (48%) [14]. Kovacs et al. observed N-cadherin expression in 30% of infiltrating cancers and 40% of DCIS. In invasive tumors, there was no significant relation between N-cadherin positivity and histological grade, lesion size, lymph node status, ER/PR status, and epidermal growth factor receptor (EGFR) expression. However, among positive DCIS, there were no low-grade lesions (75% high grade, 25% intermediate). Unfortunately, due to the small number of in situ lesions, no significant conclusion could be drawn [15]. In a large study of Aleskandarany and coworkers, a cadherin switch was more frequently detected in triple negative and human epidermal growth factor receptor (HER)2-positive subtypes of invasive nonluminal breast cancer than in the luminal subtype. There were also significant differences regarding prognosis and patient survival [16]. Nagi and colleagues reported N-cadherin expression in 76% of micropapillary cancers with high propensity for lymphatic infiltration, whereas the other types of cancer demonstrated an expression of only 52%. Staining scores were higher in invasive tumors compared to DCIS, and this effect was more dramatic in micropapillary carcinomas. The authors concluded that N-cadherin contributed to breast cancer progression and was associated with tumor aggressiveness and metastatic potential [17]. Choi et al. demonstrated a significantly higher expression of N-cadherin in invasive cancers than in pure DCIS (8.7% vs. 3.3%). Similarly, the loss of E-cadherin and upregulation of the other EMT biomarkers, such as β-catenin and smooth muscle actin, was observed [18]. In the meta-analysis of Luo et al., N-cadherin expression was found to be a useful predictor of unfavorable overall survival [19].

In the present study, the relation between N-cadherin and postoperative invasion was not significant, but close to the limit. The lack of statistical significance could have been caused by an insufficient proportion of N-cadherin positive tumors required to obtain robust statistics. The small number of N-cadherin-positive lesions may have been responsible for the relatively low rate of postoperative upgrading in the current study (16%) when compared to other series, including our previous report [20]. This could also have been influenced by the fact that we studied cases with typical clinical and radiological features (nonpalpability, microcalcifications without mass, absence of other findings on imaging (e.g., architectural distortion, asymmetric density)) of early DCIS limited to intraductal proliferation and rarely associated with the presence of an infiltrating component.

The risk of final invasion was significantly and independently related to both EMT triggers, Snail1 and SPARC. Snail1 displays a broad spectrum of biological functions: Regulation of cell movement and adhesion, cell proliferation and survival, immune suppression, and generation of stem cell properties [21]. Snail1 triggers EMT by direct repression of the transcription of the E-cadherin gene [22]. Since it is an E-cadherin repressor inducing a cadherin switch, its overexpression is a step before the acquisition of N-cadherin. Therefore, it is not surprising that during the very early phase of ductal cancer progression, as was the case in our study, expression of Snail1 better determined the risk of invasion than N-cadherin. Although Snail1 is normally absent in healthy mammary epithelial cells, it often becomes activated during breast cancer progression and is observed in about 80% of microdissected human invasive ductal cancers [23,24]. The analysis of tumor development using the MMTV-PyMT (Mouse Mammary Tumor Virus – Polyomavirus Middle T-antigen) transgenic model of mammary tumor formation, which mirrors the multistep progression of human breast cancer, demonstrated that Snail1 expression was related to potent EMT activation and acquisition of aggressive features [24]. Snail1 is closely associated with a tumor-initiating cell phenotype that is responsible for metastatic dissemination and clinical relapse in a variety of cancers [24,25,26]. In MMTV-PyMT carcinoma-derived pB1.3G cells and MDA-MB-231 (M.D. Anderson Metastatic Breast cancer cell line established from a pleural effusion of a 51-year-old female with highly aggressive and poorly differentiated triple-negative ductal cancer) human breast cancer cells, the knockdown of Snail1 attenuates primary tumor growth, strongly suppresses its metastatic spreading, and induces the acquisition of epithelial traits in a reverse epithelial-mesenchymal transition process [24]. Moreover, Snail1 knockdown significantly suppresses tumor initiation in most human breast cancer cell lines [24].

There are several challenges in reconstructing and analyzing an EMT regulatory network [27]. EMT, at the tissue level, can be recognized by the detection of the downregulation of specific epithelial biomarkers and the upregulation of specific mesenchymal biomarkers. However, the acquisition of a full mesenchymal state is rarely detected in vivo. This is due to various cells being at different stages of EMT at any given time [27,28]. The expression of EMT markers is temporally and spatially coordinated through the activation of an entire network of transcription factors that initiates and orchestrates the EMT process [27,28,29]. Morphological changes during EMT reflect the actuation of a specific molecular program, activated by juxtacrine and paracrine signals derived from the tumor microenvironment, that induces hierarchical, multilayer signaling networks [27,30]. EMT regulation by the microenvironment is extremely complex, resulting in a heterogeneous activation of several cellular pathways [27]. Numerous regulators involved in the activation of EMT have been identified. SPARC, as a key modulator of the local environment, is one such mediator. It regulates cellular differentiation, proliferation, and migration; influences cell polarity, shape, and attachment; has an impact on the activity of growth factors and membrane permeability; and is involved in stromal remodeling by regulating the expression of extracellular matrix metalloproteinases as well as collagen fibrillogenesis and deposition [31,32]. In the specific tumor microenvironment, SPARC may facilitate the degradation of the basement membrane barrier and contribute to the conversion from in situ to infiltrating cancer [24]. Our findings support the notion that gaining invasive properties is a complex process with a multidirectional cross-interaction of tumor cells and stroma.

In multivariate analysis, the presence of a postoperative infiltrating component was better predicted by preoperative SPARC than Snail1: However, both factors were significant. Separate investigations resulted in invasion risk stratification from 3% (negative) through 38% (positive) to 73% (strong expression) for SPARC, and from 3% (negative) through 25% (positive) to 55% (strong expression) for Snail1 [11,12]. Complex analysis of their expression more accurately reflected the process of gaining invasive capacity during the very early steps of cancer progression. SPARC/Snail1 expression profiles defined the subtypes of DCIS with significantly different risks of final invasion. It allowed for the identification of completely indolent (no risk of invasive component) and extremely aggressive (66–100% risk of invasive component) subtypes of DCIS. A clinical implication of this is the possibility of a more tailored approach and selective surgery. Sentinel node biopsy during breast surgery can be considered in high-risk patients. In contrast, it can be avoided in low-risk women. Moreover, in selected cases, indications for breast resection can be redefined (i.e., in double-negative patients without a visible residual postbiopsy lesion, a close radiological follow-up may be a valuable option).

With regard to the invasion-metastasis cascade, the transition from an in situ to an infiltrating phenotype is an essential step in tumor metastasis. However, the question of whether the acquisition of invasive propensity by DCIS can really predict the course of disease remains open. On the one hand, significant correlations between the expression of SPARC and unfavorable clinical outcomes and a worse prognosis in DCIS has been observed [33]. Significant associations between Snail1 expression and poor overall and disease-free survival has also been reported [34]. On the other hand, a sequential model of breast cancer progression is sometimes challenged. Narod and Sopik questioned the paradigm that breast cancer passes through several stages and claimed that invasion is not a necessary step for metastasis. They proposed an alternate theory, a parallel model wherein there is a small pool of cancer stem cells that have metastatic potential and synchronously disseminate through several routes: To the breast tissue, to the lymph nodes, and to distant organs [35]. According to this theory, cancer cells disseminating to the breast rise and make up the bulk of the tumor mass, but they are not a source of distant metastases. However, since there is still no equivocal evidence supporting either of the progression theories, some emphasize that there are probably still other unknown cancer transition pathways and that numerous mechanisms may coexist [36].

The lack of analysis of E-cadherin was a weakness of our study. N-cadherin expression was found in relatively few cases and a concomitant investigation of E-cadherin could better reflect a cadherin switch. However, since Snail1 is a direct repressor of E-cadherin, its expression and E-cadherin downregulation are closely related to each other. Therefore, the likelihood that the analysis of E-cadherin expression, in addition to Snail1, could have improved the extremely significant predictive value of Snail1 expression in our series is disputable. Snail1 is a transcription factor, and its expression is expected to be predominantly localized in the nucleus. Surprisingly, we observed exclusively cytoplasmic staining. This is not easy to explain and has been thoroughly discussed elsewhere [12]. Briefly, nuclear expression is considered better than cytoplasmic expression or Snail1 mRNA at predicting Snail1 activity, because this transcription factor is the subject of post-translational modifications that influence its stability and localization [37,38,39]. However, in some investigations, both cytoplasmic and nuclear expression have been found [14,39]. In other studies, similarly to us, predominantly or even exclusively cytoplasmic Snail1 reactivity was observed in preinvasive and invasive breast and colorectal cancer [40,41,42]. Some claim that since Snail1 displays a broad spectrum of biological functions, a subcellular localization in the cytoplasm may not be definitively associated with a total loss of its function [42]. In addition, there are probably several more Snail1-regulated processes waiting to be discovered [21].

Our study also had some other important limitations. First, this was just an observational study, and no comparison with other EMT biomarkers was performed. Second, assessment of immunohistochemical staining is always more or less related to the limited intra- and inter-observer reproducibility. Third, some findings were based on the comparison of small groups. Even if the results were significant, their statistical power was low, and therefore it was difficult to draw any conclusive statements. Fourth, it was a single-institution case series. Taking everything into account, one cannot be sure that our results will be repeatable in a different setting.

To the best of our knowledge, this recent report is the first study addressing the predictive value of the combined expression of EMT biomarkers during the very early steps of breast cancer progression (i.e., a transition from in situ to invasive disease) performed on specimens from a preoperative stereotactic biopsy. We successfully identified the different subtypes of DCIS with varying infiltrating potential, from an indolent proliferation to an extremely aggressive lesion, which allows for more individual postbiopsy decision making and a better-designed therapy.

## 4. Materials and Methods

### 4.1. Patients

We studied 209 consecutive patients with pure DCIS diagnosed via percutaneous stereotactic vacuum-assisted breast biopsy due to suspicious microcalcifications in the years 2004–2014. All of them had breast imaging reporting and data system (BIRADS) category 4 or 5 microcalcifications without a mass or architectural distortion, lack of invasion, or microinvasion (≤1 mm in the longest diameter) on a postbiopsy pathological examination, and also an absence of any other breast malignancy or borderline lesion. Biopsies were completed under digital mammography guidance using a designated prone table unit (Mammotest Plus/S, Fisher Imaging, Denver, CO, USA) with a 10-G needle (EnCore Breast Biopsy System, SenoRx Inc., Irvine, CA, USA or EnCore Enspire Breast Biopsy System, C.R. Bard Inc., Tempe, AZ, USA). All procedures were performed by a single breast-dedicated radiologist (PK) at the same breast care unit and according to the same standardized protocol in order to assure quality control. The technical details of the biopsy as well as a description of the clinical factors, mammographic presentation, and histological features have already been presented [11]. In each case informed consent was obtained. The study was conducted according to the Declaration of Helsinki principles and approved by the Independent Ethics Committee of the Wroclaw Medical University (UMED KB-376; 19 Oct 2010) and Institutional Review Board (NDOK/668; 6 August 2012).

### 4.2. Immunohistochemistry

**Figure 1:**
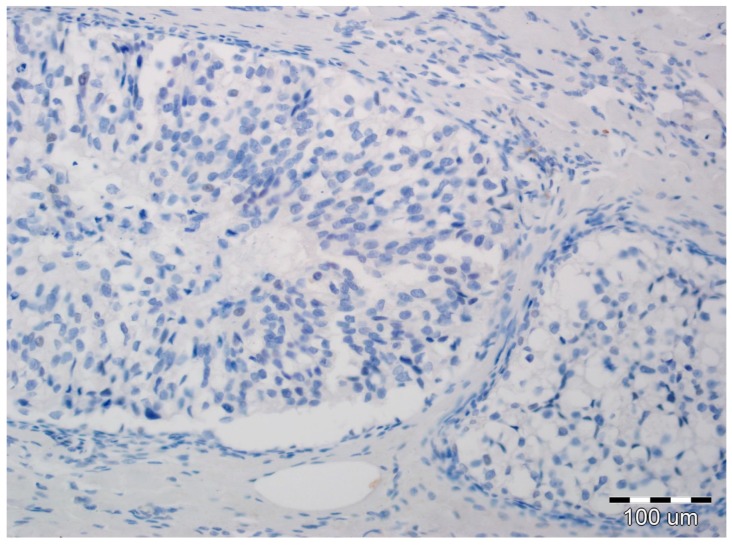
Ductal carcinoma in situ with lack of N-cadherin expression in luminal epithelial cells and the stromal compartment (hematoxylin, ×200).

**Figure 2:**
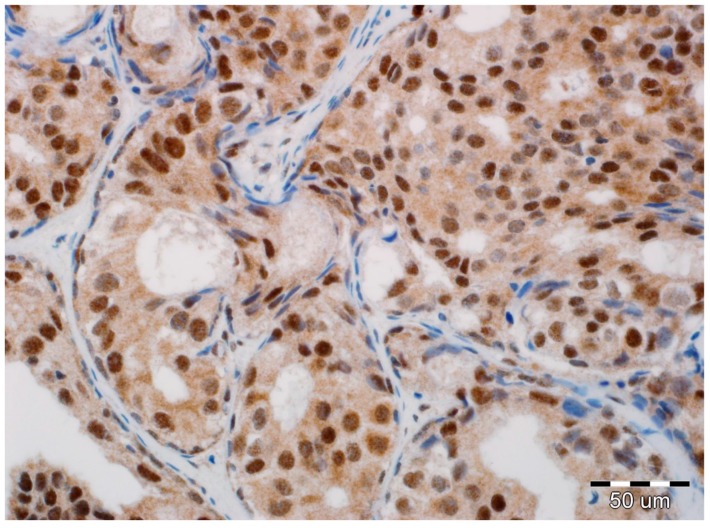
Positive expression of N-cadherin in luminal epithelial cells of ductal carcinoma in situ (DCIS). No reaction in stromal fibroblasts and myoepithelial cells (hematoxylin, ×400).

All slides of formalin-fixed and paraffin-embedded tissue specimens obtained from biopsies were hematoxylin-eosin-stained and then examined by two board-certified pathologists experienced in breast cancer (A.H., P.D.). All immunohistochemical evaluations were performed without knowledge of tumor and patient characteristics. In all cases, pure DCIS without an invasive or microinvasive component was reported. Expression of EMT biomarkers was assessed in luminal epithelial cells of DCIS. In each case, immunoreactivity was scored by the two study pathologists, without conflicting observations. Immunohistochemical determination of N-cadherin expression was completed using a mouse monoclonal antibody (NC-17; catalog code: NBP1-41353; dilution: 1:100, Novus Biologicals LCC, Littleton, CO 80120, USA). It was performed on 4-µm-thick paraffin sections mounted on silanized slides (code number S 3003, DAKO, Glostrup, Denmark), which were then subjected to deparaffinization, rehydration, and heat-induced epitope unmasking performed using PT Link, with incubation in EnVision™. Target Retrieval Solution for 20–40 min at 97 °C. Autostainer Link was used to perform an immunological test using the detection reagent DakoEnVision™ FLEX/HRP (SM802). Expression was assessed as negative in cases of no staining (Figure 1) or only staining of up to 20% of cells, and positive when more than 20% of the analyzed cells were stained (Figure 2). Due to the small number of positive cases, we did not stratify the reactivity into weak, intermediate, and strong staining.

**Figure 3:**
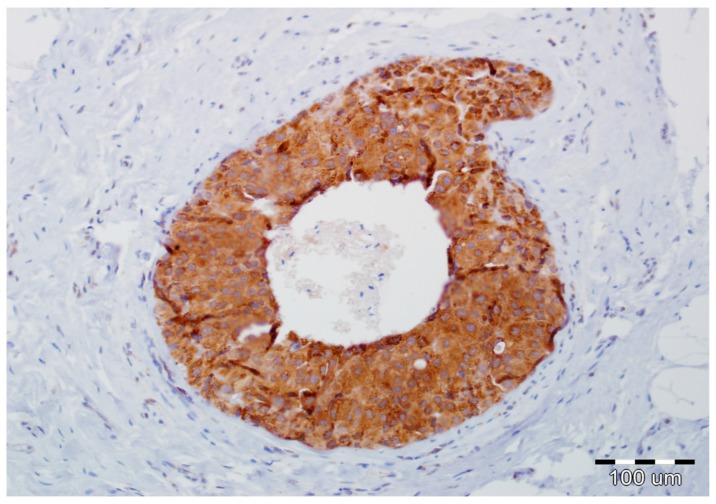
Strong expression of Snail1 in DCIS luminal epithelial cells and lack of its reactivity in myoepithelial cells and stromal fibroblasts (hematoxylin, ×200).

**Figure 4:**
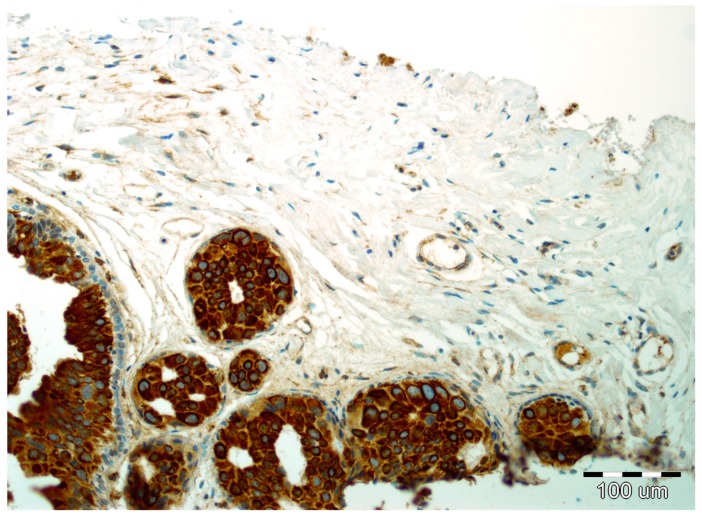
Strong expression of SPARC in DCIS luminal epithelial cells and focal reactivity in the stromal compartment with lack of reactivity in myoepithelial cells (hematoxylin, ×200).

Details of the immunohistochemistry of SPARC and Snail1 have been presented elsewhere [11,12]. For both, a semiquantitative method was used to evaluate the expression as negative (0, no staining), weak (1, either diffuse weak staining or strong staining in <10% of analyzed cells), intermediate (2, either diffuse intermediate staining or strong staining in ≥10% to 30% of analyzed cells), and strong (3, defined as strong staining of >30% of analyzed cells). Strong expression of Snail1 and SPARC in DCIS luminal epithelial cells is presented in Figure 3 and Figure 4, respectively.

### 4.3. Statistical Analysis

Data was collected prospectively and entered into a computer database. Upgrading to invasive disease on final pathology of the postoperative specimen was calculated. A Kruskal–Wallis one-way analysis of variance was used to verify the relation between the expression of EMT biomarkers and categorical variables, whereas a Spearman’s correlation coefficient was used for the continuous variables. The association between postoperative invasion and preoperative features was assessed using a chi-square test for categorical variables and a nonparametric Wilcoxon test for continuous variables. Multivariate analysis was performed with a multiple logistic regression. A *p*-value of 0.05 was considered significant. Statistical analysis was performed by a professional statistician (P.B.) with R-software ver. 3.2 (free environment for statistical computing and graphics).

## 5. Conclusions

The biology of DCIS is still not well understood. It is a heterogenic disease, and a significant proportion of these lesions will never lead to invasive breast cancer [43]. Therefore, risk stratification is essential to make better-informed clinical decisions [43,44]. We demonstrated that SPARC and Snail1 were better predictors of postoperative invasion than N-cadherin. Moreover, using their combined expression profiles, indolent and very aggressive subtypes of DCIS could be preoperatively identified, which can help to tailor optimal management. Since DCIS represents an increasing proportion of newly diagnosed neoplastic breast lesions, it is vital to prevent the overtreatment of patients in cases of harmless DCIS, but concurrently to provide optimal therapy for potentially hazardous DCIS [44].

## Figures and Tables

**Table 1:** Baseline characteristics.

Variable	Results
Patient age	
Median/range	59/41–88
Mean ± SD	58.4 ± 7.9
Microcalcification type, *n* (%)	
Powdery	70 (34)
Crushed stone-like	107 (51)
Casting-type	32 (15)
Microcalcification distribution, *n* (%)	
Cluster	145 (69)
Group	44 (21)
Regional	20 (10)
Nuclear grade, *n* (%)	
Low	110 (52)
Intermediate	66 (32)
High	33 (16)
Comedonecrosis, *n* (%)	
Absent	93 (45)
Present	116 (55)
N-cadherin expression, *n* (%)	
Negative	192 (92)
Positive	17 (8)
Snail1 expression, *n* (%)	
Negative	80 (38)
Positive	129 (62)
Weak	57 (27)
Intermediate	43 (21)
Strong	29 (14)
SPARC expression, *n* (%)	
Negative	130 (62)
Positive	79 (38)
Weak	33 (16)
Intermediate	20 (10)
Strong	26 (12)

**Table 2:** Significant correlation between N-cadherin, Snail1, and secreted protein acidic and rich in cysteine (SPARC) expression; *n* (%).

	N-Cadherin		SPARC		Snail1	
	Negative	Positive	Negative	Positive	Negative	Positive
N-cadherin						
Negative	-	-	124 (65)	68 (35)	78 (41)	114 (59)
Positive	-	-	6 (35)	1 (65)	2 (12)	15 (88)
SPARC						
Negative	124 (95)	6 (5)	-	-	66 (51)	64 (49)
Positive	68 (86)	11 (14)	-	-	14 (18)	65 (82)
Snail1						
Negative	78 (97)	2 (3)	66 (82)	14 (18)	-	-
Positive	114 (88)	15 (12)	64 (49)	65 (51)	-	-

**Table 3:** Association between preoperative features and final invasion on surgical specimens.

Variable	Postoperative Invasion		Univariate Analysis *p*	Multivariate Analysis *p*
	Absent *n* (%)	Present *n* (%)		
Microcalcifications type				
Powdery	59 (84)	11 (16)	0.645	-
Crushed stone-like	91 (85)	16 (15)		
Casting-type	25 (78)	7 (22)		
Microcalcifications distribution				
Cluster	123 (85)	22 (15)	0.771	-
Group	36 (76)	8 (24)		
Regional	16 (80)	4 (20)		
Nuclear grade				
Low	95 (86)	15 (14)	0.417	-
Intermediate	52 (79)	14 (21)		
High	28 (85)	5 (15)		
Comedonecrosis				
Absent	80 (86)	13 (14)	0.520	-
Present	95 (82)	21 (18)		
N-cadherin expression				
Negative	162 (84)	30 (16)	0.061	-
Positive	13 (76)	4 (24)		
Snail1 expression				
Negative	78 (97)	2 (3)	<0.0001	0.007
Positive	97 (75)	32 (25)		
Weak	52 (91)	5 (9)		
Intermediate	32 (74)	11 (26)		
Strong	13 (45)	16 (55)		
SPARC expression				
Negative	126 (97)	4 (3)	<0.0001	<0.0001
Positive	49 (62)	30 (38)		
Weak	32 (97)	1 (3)		
Intermediate	10 (50)	10 (50)		
Strong	7 (27)	19 (73)		
Patient age				
Median (range)	59 (41–88)	58.5 (43–75)	0.980	-

**Table 4:** Invasion rate with regard to SPARC and Snail1 immunoreactivity.

SPARC Expression *n* (%)	Snail1 Expression *n* (%)			
	Negative	Weak	Intermediate	Strong
Negative	0 (0/66)	3% (1/38)	10% (2/21)	20% (1/5)
Weak	0 (0/9)	0 (0/9)	0 (0/9)	17% (1/6)
Intermediate	0 (0/3)	33% (3/9)	75% (3/4)	100% (4/4)
Strong	100% (2/2)	100% (1/1)	66% (6/9)	71% (10/14)

## Abbreviations

DCIS	Ductal carcinoma in situ
EMT	Epithelial-mesenchymal transition
SPARC	Secreted protein acidic and rich in cysteine
BIRADS	Breast imaging reporting and data system
ER	Estrogen receptor
PR	Progesterone receptor
EGFR	Epidermal growth factor receptor
HER2	Human epidermal growth factor receptor 2
